# Self-reported cannabis use to manage opioid withdrawal symptoms and reductions in opioid use among people who use unregulated opioids: a cross-sectional analysis

**DOI:** 10.1186/s42238-026-00458-0

**Published:** 2026-06-24

**Authors:** Erica McAdam, Hudson Reddon, M Eugenia Socías, Stephanie Lake, Kanna Hayashi, Kora DeBeck, Zach Walsh, M-J Milloy

**Affiliations:** 1 British Columbia Centre On Substance Use, Vancouver, BC Canada; 2 Interdisciplinary Studies Graduate Program, University of British Columbia, Vancouver, BC Canada; 3 Department of Medicine, University of British Columbia, 2775 Laurel Street, Vancouver, BC V6Z 1Y6 Canada; 4 Department of Psychiatry and Biobehavioral Sciences, UCLA Center for Cannabis and Cannabinoids, Jane and Terry Semel Institute for Neuroscience and Human Behavior, University of California, Los Angeles, CA USA; 5 Faculty of Health Sciences, Simon Fraser University, Burnaby, BC Canada; 6 School of Public Policy, Simon Fraser University, Vancouver, BC Canada; 7 Department of Psychology, University of British Columbia, Kelowna, BC Canada

**Keywords:** Cannabis, Opioids, People who use drugs, Opioid withdrawal, Harm reduction

## Abstract

**Background:**

Opioid withdrawal is a significant challenge for people seeking to reduce or eliminate opioid use, and unmanaged withdrawal increases the risk of relapse and overdose. Using cannabis to manage opioid withdrawal has been reported by people who use opioids, yet it is not clear whether this leads to reductions in opioid use. Moreover, because pain is prevalent among people who use unregulated opioids (PWUO) and may contribute to ongoing opioid use, the effects of cannabis use to manage withdrawal symptoms may differ among individuals experiencing moderate to severe pain. We investigated the relationship between cannabis use to manage unregulated opioid withdrawal and self-reported reductions in opioid use among PWUO.

**Methods:**

Data were derived from a cross-sectional questionnaire administered to cannabis-using PWUO in Vancouver, Canada, between December 2019 and November 2021. Multivariable logistic regression estimated the associations between cannabis use for opioid withdrawal and self-reported reductions in opioid use. A sub-analysis explored if these associations varied among participants living with and without moderate to severe pain.

**Results:**

Among 197 participants, 89 (45.2%) reported cannabis use to manage symptoms of opioid withdrawal in the past six months. In multivariable analysis, cannabis use for opioid withdrawal was significantly associated with self-reported reductions in opioid use (adjusted Odds Ratio [AOR] = 2.16, 95% Confidence Interval [CI]: 1.13–4.19) in the same time period. In a sub-analysis, this association was only significant among participants with moderate to severe pain (AOR = 6.55; 95% CI: 2.44–19.63).

**Conclusions:**

We observed a significant association between self-reported use of cannabis to manage unregulated opioid withdrawal and reductions in opioid use among cannabis-using PWUO living with pain. Aligned with other studies, these findings support conducting experimental trials of cannabinoids to support individuals experiencing opioid withdrawal and living with pain.

## Introduction

Canada and the United States continue to experience high rates of morbidity and mortality arising from exposure to the unregulated drug supply driven by the replacement of opiates (e.g., heroin, morphine) with synthetic opioids such as fentanyl and its analogues, which have been clandestinely produced or diverted from licit supplies (Fischer et al. 2020, 2019; Zoorob 2019; Han et al. 2019; BC Coroners Service Death Review Panel 2023, 2022). In Canada, the rate of opioid-associated deaths increased from 7.8 per 100,000 population in 2016 to 17.6 per 100,000 population in 2024 (Government of Canada 2024). The western Canadian province of British Columbia (BC) has among the highest toxic drug mortality rates, increasing from 20.5 per 100,000 population in 2016 to 46.7 in 2023. In 2024, drug toxicity (or overdose) deaths were the leading cause of death in those aged 10–59 years in the province (BC Coroners Service 2024). There is an urgent need to scale up effective evidence-based interventions (such as low-barrier medications for opioid use disorder, community-distributed naloxone, and supervised consumption spaces) as well as identify new approaches to reduce morbidity and mortality among people who use drugs (PWUD) at high risk of fatal overdose.

Using cannabis has emerged as a potential harm reduction strategy in the management of unregulated opioid use, including for symptoms of opioid withdrawal (Lucas 2017; Wiese and Wilson-Poe 2018; Aquino et al. 2022; Mok et al. 2021, 2023; Reddon et al. 2023; Socías et al. 2021). Pre-clinical, observational, and experimental studies have suggested that cannabis use may modulate opioid withdrawal and alleviate symptoms such as pain, anxiety, tremors, and insomnia (Aquino et al. 2022, 2023; Lofwall et al. 2016; Hurd et al. 2019; Bisaga et al. 2015). Experimental studies have demonstrated that the non-intoxicating cannabinoid cannabidiol (CBD) can reduce opioid cravings and anxiety—both core features of opioid withdrawal that are strongly linked to resuming opioid use highlighting plausible mechanisms through which cannabinoids may support reductions in opioid use (Hurd et al. 2019; Kudrich et al. 2021). Qualitative studies have also documented self-reported use of cannabis among PWUD to ease opioid withdrawal symptoms (Boyd et al. 2017; Meacham et al. 2022; Paul et al. 2020). Despite the availability of effective medication-based treatments for withdrawal and opioid use disorder in Canada, including buprenorphine/naloxone and methadone, there are numerous barriers to access, including provider stigma, out-of-pocket costs, criminalization, and systemic gaps in service provision, for example, dispensation locations that are few and far between for people living in rural or remote settings (Vipler et al. 2018; Frank et al. 2021; Davis and Carr 2019; Pijl et al. 2022; Gallant et al. 2025; Bruneau et al. 2018; BC Centre on Substance Use, BC Ministry of Health, BC Ministry of Mental Health and Addictions 2023; Madras et al. 2020; Hall et al. 2021; Sharma et al. 2017; Pilarinos et al. 2022, 2022). In addition, some individuals may prefer non-opioid-based approaches or struggle with side effects and daily adherence challenges associated with opioid agonist therapy (OAT) (Wiese and Wilson-Poe 2018). A recent population-based retrospective cohort study in BC found that approximately eight-in-ten patients starting methadone or buprenorphine/naloxone had discontinued treatment at 24 months post-initiation (Nosyk et al. 2024). Patient engagement on OAT is often limited by inadequate dosing, which can limit treatment effectiveness and contribute to ongoing withdrawal symptoms, cravings, unregulated opioid use, and potential discontinuation (D’Aunno et al. 2014; Lake et al. 2023). Experiencing opioid withdrawal can be a significant barrier for people seeking to reduce or eliminate opioid use (Kosten and Baxter 2019). Moreover, unmanaged withdrawal increases the risk of resuming unregulated opioid use and other drug-related harms, including HIV transmission and disease progression and risk of accidental overdose, as individuals who abruptly discontinue use of opioids may experience heightened cravings and reduced tolerance (Kudrich et al. 2021; Wakeman et al. 2020; Wines et al. 2007; Torres-Lockhart et al. 2022). Expanding opioid withdrawal management options—including non-traditional approaches—may support individuals in reducing opioid use while minimizing harm.

Much of the existing literature on cannabis and opioid withdrawal focuses on whether cannabis effectively alleviates withdrawal symptoms with limited attention paid to possible effects on opioid use. Thus, we aimed to fill this gap by investigating the relationship between self-reported cannabis use to manage unregulated opioid withdrawal symptoms and self-reported changes in opioid use during periods of cannabis use among people who use unregulated opioids (PWUO) in Vancouver, Canada, independent of engagement with OAT. In addition, informed by prior research documenting the importance of chronic pain in moderating these effects and the high prevalence of uncontrolled chronic pain among PWUO (Reddon et al. 2023; Lake et al. 2019; Okusanya et al. 2020; Lucas et al. 2020; Kitchen et al. 2025; Voon et al. 2014, 2017), we conducted a sub-analysis among participants reporting living with and without moderate to severe pain. Chronic pain may modify this relationship because unmanaged chronic pain is a driver of ongoing opioid use and withdrawal severity, and cannabinoids have demonstrated analgesic properties that modulate pain perception (Kitchen et al. 2025; Voon et al. 2014; Ware et al. 2022). Accordingly, we used an interaction test and stratified analysis to investigate if the potential relationship between using cannabis to manage opioid withdrawal and reductions in opioid use is modified by the presence of pain.

## Methods

### Data sources and participants

To address our objectives, we collected data from three open prospective cohorts of people who use drugs (PWUD) in Vancouver, Canada: the Vancouver Injection Drug Users Study (VIDUS), the At-Risk Youth Study (ARYS), and the AIDS Care Cohort to Evaluate exposure to Survival Services (ACCESS). Details of these studies and their harmonized procedures have been previously described (Strathdee et al. 1997; Wood et al. 2006; Milloy et al. 2015). Briefly, to be eligible for enrollment, participants in all three cohorts must report unregulated/illicit drug use in the past month (other than or in addition to cannabis, alcohol, or tobacco), live in the Greater Vancouver Regional District, and provide written informed consent. The VIDUS cohort includes people who are 18 years or older, have injected drugs within the past month at baseline, and tested seronegative for HIV at baseline. ACCESS includes participants who are 18 years or older and are living with HIV as determined via serology. Finally, the ARYS cohort includes people between the ages of 14–26 at baseline who are street-involved, defined as being without stable housing or utilizing services for youth who are experiencing homelessness within the past month (Debeck et al. 2013).

At baseline and biannually thereafter, participants complete an interviewer-administered questionnaire that collects data on demographics, substance use patterns and associated risks, income generation activities, and health and social service engagement, among others. All participants received a $40 CAD honorarium at each study visit during the current study period. From December 2019 to November 2021, participants from all three cohorts who reported any form of cannabis use in the past six months were invited to complete a supplementary questionnaire in addition to their routine study interview follow-up. The supplementary cannabis questionnaire collected data on the frequency of cannabis use, route(s) of administration, cannabis source, motivations for use (e.g., intoxication, recreation, pain relief, substitution, spiritual/creativity), and perceived effects of cannabis use on other substance use (e.g., substitution vs. complementarity). Participants who completed the supplementary cannabis questionnaire were given an additional $40 CAD honorarium. All data collection was suspended during the initial wave of the COVID-19 pandemic (i.e., March 17 to July 16, 2020); from July 17, 2020 to February, 1, 2022, data collection for the cohorts and cannabis supplement was completed remotely via telephone or videoconferencing with loaner cellphones and a semi-private space provided to any participant as needed. Data collection procedures during the COVID-19 pandemic have been described in detail and published elsewhere (McAdam et al. 2022). Ethical approval for the cohorts was obtained from the University of British Columbia/Providence Health Care, and Simon Fraser University Research Ethics Boards (H22-03285,H05-50233), and approval for the cannabis supplement was obtained from the University of British Columbia/Providence Health Care Research Ethics Board (H19-01102).

### Study sample

The analytic sample for the present study included all participants who completed the supplementary cannabis questionnaire (therefore, all participants who reported cannabis use in the past six months), had non-missing data for the main outcome, primary explanatory, and secondary variables (described below), and also reported any unregulated opioid use in the past six months, specifically, reporting any use of fentanyl, unspecified opioids, heroin, and/or other unregulated opioids, including use of non-medical prescription opioids.

### Study variables

Our outcome of interest was self-reported reductions in opioid use during periods of cannabis use, based on the survey question “When I use cannabis, I don’t need to use as much of the opioids that I am taking.” Response options were based on a five-point Likert scale ranging from *strongly disagree* to *strongly agree*. Participants who responded, *strongly agree* or *somewhat agree* were coded as “yes” and participants who responded *strongly disagree*, *somewhat disagree* or *neither agree nor disagree* were coded as “no.” The primary explanatory variable of interest was self-reported use of cannabis to manage symptoms of illicit/unregulated opioid withdrawal (yes vs. no), based on the following two questions: 1) “In the last six months, have you ever used cannabis to reduce symptoms of withdrawal of other drugs?” (yes vs. no); and 2) “If yes, for what other drugs?” (illicit opioids vs. prescription opioids, other prescription (benzos, steroids), stimulants, alcohol, tobacco, or other). Participants who answered “yes” to the first question and selected “illicit opioids” for the second question were coded as those who “used cannabis for opioid withdrawal” (i.e., “yes”). Participants who answered “no” to the first question, in addition to participants who answered “yes” to the first question but selected any other type of drug other than illicit opioids, were coded as those who “did not use cannabis for opioid withdrawal” (i.e., “no”). Importantly, these variables capture related but distinct constructs: the explanatory variable reflects intentional cannabis use specifically to manage opioid withdrawal symptoms, whereas the outcome reflects participants’ perceived changes in opioid use during periods of cannabis use, regardless of their original motivation for cannabis use.

We also selected a range of variables we hypothesized could affect the relationship between using cannabis to manage symptoms of opioid withdrawal and reductions in opioid use during periods of cannabis use, based on a priori understanding and previous studies investigating cannabis substitution for opioid use and cannabis use as harm reduction (Mok et al. 2023; Reddon et al. 2023; Lake et al. 2019; Kvamme et al. 2021; Lucas et al. 2019; Reiman et al. 2017). These were: 1) age (per year older),2) self-identified gender (woman, transgender, Two-Spirit, and those who preferred to self-describe their gender identity vs. man); 3) race/ancestry (Black, Indigenous, or Person of Colour vs. white); 4) experiencing homelessness (yes vs. no); 5) use of free cannabis distribution programs (which referred to community-based efforts offering small amounts of unregulated cannabis to individuals as a harm reduction intervention; yes vs. no); 6) current pain severity at time of interview (moderate to severe vs. none or slight), based on Euroqol EQ-5D pain scale (Obradovic et al. 2013; Zanden et al. 2006); 7) cannabis use frequency (< weekly vs. at least weekly, at least once a day); 8) drug or alcohol treatment (defined as having engaged with any drug or alcohol treatment programme, including opioid agonist therapy, detox/withdrawal management, recovery house, treatment centres, counselling, Narcotics Anonymous/Cocaine Anonymous/Alcoholics/Anonymous/Self-Management and Recovery Training (SMART), out-patient treatment, or drug treatment court; yes vs. no); and 9) experienced barriers to accessing addiction treatment (yes vs. no). Except for time-fixed variables (i.e., race and ancestry) all variables referred to the six-month period prior to the interview.

### Statistical analyses

As a first step, characteristics of the study sample for the variables listed above, in addition to a variable on opioid use frequency (≥ daily vs. < daily), were stratified by the primary explanatory variable, self-reported use of cannabis to manage symptoms of opioid withdrawal. Differences in characteristics between participants who reported using cannabis for opioid withdrawal versus those who did not were investigated using chi-square test and Fisher exact test (when chi-square assumptions were not met) for categorical variables and T-test for continuous variables. Bivariable and multivariable logistic regression models were then fit to estimate the unadjusted and adjusted relationships between the main outcome (self-reported reductions in opioid use during periods of cannabis use) and the primary explanatory variable (self-reported use of cannabis to manage symptoms of opioid withdrawal). The nine variables listed above were included as covariates in the multivariable model. A sub-analysis was conducted among people living with moderate to severe pain. First, an interaction term was included in the final multivariable model between the primary outcome variable (cannabis use to manage symptoms of unregulated opioid withdrawal) and the pain variable. After observing that the interaction term was significant, we ran two different multivariable logistic regression models using the same steps above, on two different sub-samples: one among participants living with none or slight pain and the other among participants living with moderate to severe pain. All statistical analyses were performed using R version 4.4.0 (The R Foundation for Statistical Computing, Vienna, Austria), and all tests of significance were two-sided with a significance threshold of *p* < 0.05.

## Results

Overall, 197 participants from the VIDUS (*n* = 90, 45.9%), ACCESS (*n* = 45, 23.0%), and ARYS (*n* = 61, 31.1%) cohorts completed the supplementary cannabis questionnaire, reported any unregulated opioid use in the past six months, and had non-missing data for variables of interest. (Eight [3.9%] participants were removed due to missing data). Among the study sample, the median age at the earliest interview was 40 years (interquartile range: 29.5–53.6 years old), 70 (35.7%) identified as being Black, Indigenous or a person of colour, and 66 (33.7%) participants identified as women, 2 (1.0%) as transgender, 3 (1.5%) as Two-Spirit, and 2 (1.0%) self-described their gender identity. Overall, 89 (45.2%) participants reported using cannabis in the last six months to manage symptoms of unregulated opioid withdrawal, and among these participants, 59 (66.3%) reported decreasing their opioid use during periods of cannabis use.

Characteristics of the study sample stratified by reported use of cannabis to manage symptoms of unregulated opioid withdrawal are presented in Table 1. Participants who reported cannabis use to manage unregulated opioid withdrawal symptoms were more likely to be younger, engage in at least daily opioid use, less likely to use cannabis on a less than weekly basis, and more likely to report reductions in opioid use during periods of cannabis use (all *p* < 0.05), compared to participants who did not report using cannabis to manage opioid withdrawal.

**Table 1:** Characteristics of 197 people who use unregulated opioids stratified by self-reported use of cannabis to manage opioid withdrawal symptoms, Vancouver, Canada, December 2019-November 2021

		**Use cannabis for opioid withdrawal**		
**Characteristic**	**Total** n (%) 197 (100)	**Yes** n (%) 89 (45.2)	**No** n (%) 108 (54.8)	***p- *value**
Age				
Median	40.1	36.0	47.3	< 0.001
IQR (Q1-Q3)	(29.5-53.6)	(27.5–46.1)	(32.2-57.9)	
Gender				
Women, transgender, Two-Spirit, or self-described	73 (37.1)	36 (40.4)	37 (34.3)	0.455
Men	124 (62.9)	53 (59.6)	71 (65.7)	
Race/Ancestry				
BIPOC	70 (35.5)	32 (36.0)	38 (35.2)	1.000
White	127 (64.5)	57 (64.0)	70 (64.8)	
Homelessness*				
Yes	47 (23.9)	25 (28.1)	22 (20.4)	0.273
No	150 (76.1)	64 (71.9)	86 (79.6)	
Use of free cannabis distribution programs*				
Yes	58 (29.4)	31 (34.8)	27 (25.0)	0.177
No	139 (70.6)	58 (65.2)	81 (75.0)	
Pain*				
Moderate to severe	95 (48.2)	47 (52.8)	48 (44.4)	
None or slight	102 (51.8)	42 (47.2)	60 (55.6)	0.305
Opioid use*				
> Daily	118 (59.9)	66 (74.2)	52 (48.1)	< 0.001
< Daily	79 (40.1)	23 (25.8)	56 (51.9)	
Cannabis use*				
< Weekly	48 (24.4)	13 (14.6)	35 (32.4)	0.014
At least weekly	67 (34.0)	33 (37.1)	34 (31.5)	
At least once a day	82 (41.6)	43 (48.3)	39 (36.1)	
Drug/alcohol treatment*				
Yes	144 (73.1)	68 (76.4)	76 (70.4)	0.430
No	53 (26.9)	21 (23.6)	32 (29.6)	
Barriers to drug/alcohol treatment*^a^				
Yes	7 (3.6)	4 (4.5)	3 (2.8)	0.703
No	190 (96.4)	85 (95.5)	105 (97.2)	
Reductions in opioid use				
Yes	112 (56.9)	59 (66.3)	53 (49.1)	0.022
No	85 (43.1)	30 (33.7)	55 (50.9)	

Categorical variables tested using chi square test and continuous variables tested using a t-test. ^a^ tested using Fisher exact test given small cell counts

Complete case analysis; therefore, no missing values (before removal: treatment barriers (2%); homelessness (1%); free cannabis distribution programs (0.5%); daily opioid (0.5%)

*IQR* interquartile range, *Q1* quarter 1, *Q3* quarter 3, *BIPOC* Black, Indigenous or Person of Colour

^*^Refers to activities in the 6 months prior to the interview

Results of bivariable and multivariable analyses are displayed in Table 2. In unadjusted bivariable analysis, reporting the use of cannabis to manage unregulated opioid withdrawal symptoms was significantly associated with reporting reductions in opioid use during periods of cannabis use (Odds Ratio = 2.01, 95% Confidence Interval [CI]: 1.13–3.61). In the multivariable model, after adjusting for covariates, cannabis to manage unregulated opioid withdrawal symptoms remained significantly associated with self-reported reductions in opioid use during periods of cannabis use (adjusted Odds Ratio [AOR] = 2.16, 95% CI: 1.13–4.19).

**Table 2:** Bivariable and multivariable logistic regression analyses of relationship between self-reported use of cannabis to manage opioid withdrawal symptoms and reductions in opioid use among people who use unregulated opioids in Vancouver, Canada from December 2019 to November 2021 (*n* = 197)

	**Unadjusted**		Adjusted*	
**Characteristic**	**Odds Ratio** **(95% CI**)	***p-*value**	**Odds Ratio** **(95% CI**)	***p-*value**
Age (per year older)	1.01 (0.98–1.03)	0.577	1.02 (0.99–1.04)	0.161
Gender (Women, trans, Two-Spirit or self-described vs. men)	1.14 (0.64–2.06)	0.656	1.40 (0.71–2.81)	0.337
Race/Ancestry (BIPOC vs. white)	0.78 (0.43–1.41)	0.401	0.69 (0.35–1.35)	0.275
Homelessness* (yes vs. no)	0.92 (0.48–1.79)	0.808	1.13 (0.54–2.41)	0.744
Use of free cannabis distribution programs* (yes vs. no)	1.36 (0.73–2.56)	0.340	1.07 (0.54–2.15)	0.850
Pain* (moderate/severe vs. none/slight)	1.00 (0.57–1.76)	0.998	0.89 (0.48–1.64)	0.713
Cannabis use* (< weekly vs. at least weekly)	1.49 (0.71–3.17)	0.292	1.27 (0.56–2.88)	0.560
(< weekly vs. at least once a day)	2.62 (1.27–5.51)	0.010	2.39 (1.08–5.40)	0.033
Cannabis for withdrawal* (yes vs. no)	**2.04 (1.15–3.67)**	**0.016**	**2.17 (1.14–4.19)**	**0.019**
Drug/alcohol treatment* (yes vs. no)	0.82 (0.43–1.55)	0.545	0.67 (0.32–1.34)	0.261
Barriers to drug/alcohol treatment* (yes vs. no)	0.56 (0.11–2.59)	0.452	0.52 (0.09–2.73)	0.443

*CI* confidence interval, *BIPOC* Black, Indigenous or Person of Colour

^*^Refers to activities in the 6 months prior to the interview

In the sub-analysis investigating whether the relationship between using cannabis for opioid withdrawal symptoms and reductions in opioid use is modified by the presence or absence of pain, when including an interaction term between the primary explanatory variable and pain in the fully adjusted model, the interaction term was statistically significant (*p* = 0.009). Results of the multivariable models stratified by pain are displayed in Table 3. Among 102 (52%) participants living with none/slight pain, cannabis use to manage symptoms of opioid withdrawal was not significantly associated with self-assessed reductions in opioid use (AOR = 0.70; 95% CI: 0.26–1.86; *p* = 0.479). Among 95 (48%) participants living with moderate/severe pain, cannabis use to manage symptoms of opioid withdrawal was significantly associated with self-assessed reductions in opioid use (AOR = 6.55; 95% CI: 2.44–19.63; *p* = < 0.001).

**Table 3:** Multivariable logistic regression analysis of relationship between self-reported use of cannabis to manage opioid withdrawal symptoms and reductions in opioid use among people who use unregulated opioids living with moderate to severe pain in Vancouver, Canada from December 2019 to November 2021 (*n* = 197)

	**Pain**			
	**None or Slight** **(*n* = 102, 51.8%)**		**Moderate to Severe** **(*n* = 95, 48.2%)**	
**Characteristic**	Adjusted Odds Ratio* (95% CI)	***p-*value**	Adjusted Odds Ratio* (95% CI)	***p-*value**
Cannabis for withdrawal				
(yes vs. no)	0.70 (0.26–1.86)	0.479	6.55 (2.44–19.63)	< 0.001

*CI* confidence interval

^*^Multivariable models adjusted for the following variables: age; gender; race/ancestry; homelessness; access to free cannabis distribution programs; cannabis use frequency; drug/alcohol treatment; barriers to drug/alcohol treatment

## Discussion

In this study of 197 community-recruited people who use unregulated opioids and cannabis, we found that self-reported cannabis use to manage unregulated opioid withdrawal symptoms was significantly associated with self-reported reductions in opioid use during periods of cannabis use. In sub-analyses stratified by pain, we observed that this relationship was modified by the presence of pain. Specifically, among participants experiencing moderate to severe pain, we observed a significant association between self-reported use of cannabis for opioid withdrawal and self-reported reductions in opioid use (*p* = < 0.001), whereas this relationship was not statistically significant among participants living with none or slight pain (*p* = 0.479). These findings contribute to the growing body of literature documenting how people at high risk of overdose may use cannabis as a strategy to manage their opioid use (Mok et al. 2023, 2021; Ceasar et al. 2021; Ganesh et al. 2024; Beaugard et al. 2024; Lake et al. 2020). Although prior research has examined the role of cannabis in mitigating opioid withdrawal symptoms, to our knowledge, this study is the first quantitative study to specifically investigate the relationship between intentional cannabis use for withdrawal management and changes in unregulated opioid use.

The observed association between self-reported cannabis use to manage unregulated opioid withdrawal symptoms and self-reported reductions in opioid use is biologically plausible as cellular receptor systems for opioids and cannabis are co-localized throughout the central nervous system and have been shown to produce analgesia via interaction (Wiese and Wilson-Poe 2018; Aquino et al. 2022; Scavone et al. 2013; Abrams et al. 2011). Pre-clinical, observational, and experimental studies have suggested that cannabis may modulate opioid withdrawal and alleviate symptoms (Aquino et al. 2022). A recent systematic review by De Aquino and colleagues synthesized observational and experimental studies evaluating the impacts of cannabis and THC use on symptoms of opioid withdrawal among people who use opioids (regulated or unregulated). The authors found mixed results in observational studies between cannabis exposure and alleviation of opioid withdrawal symptoms,experimental studies found more consistent mild-to-moderate effects of THC on alleviating withdrawal (Aquino et al. 2022). The authors propose that these discrepant findings between observational and experimental studies are likely a result of differences in participant characteristics, cannabis dose, and the type of opioids from which the participants are experiencing withdrawal (Aquino et al. 2022). The intersection of these factors likely moderates the association between cannabis exposure and opioid outcomes. Qualitative work also supports the findings we observed. For example, one qualitative study by Ganesh et al. among people who inject drugs in Los Angeles found that participants described using cannabis to reduce opioid use, including to manage symptoms of opioid withdrawal (Ganesh et al. 2024). Specifically, participants discuss using cannabis to manage the physical pain of withdrawal, and a number of participants also described that using cannabis to manage withdrawal also helped them reduce the frequency of opioid use (Ganesh et al. 2024). These findings are consistent with our observation that participants self-reporting cannabis use for opioid withdrawal were more likely to self-report reductions in opioid use.

Our findings may also be understood in the context of prior research supporting the potential use of cannabidiol (CBD), a non-intoxicating cannabinoid, to manage opioid withdrawal symptoms (Kudrich et al. 2021, 2024; Hurd 2017). For example, a double-blind randomized control trial conducted by Hurd et al. among drug-abstinent people living with opioid use disorder found that high doses of CBD (400 mg or 800 mg) versus placebo significantly reduced both drug cravings and anxiety following three days of administration, with effects persisting for up to one week after the final dose (Hurd et al. 2019). Anxiety is a key symptom of opioid withdrawal and has been found to increase the risk of relapse (Kudrich et al. 2021; Moradinazar et al. 2020). These findings may help contextualize the relationship observed in our study between self-reported cannabis use for withdrawal management and self-reported reductions in opioid use. Notably, doses of CBD administered in this trial likely substantially exceed the levels of CBD typically obtained through non-medical cannabis use, including cannabis products commonly used by participants in community settings from both regulated (e.g., cannabis stores) and unregulated (e.g., street-level dealers) outlets.

Our findings add to the growing observational literature describing real-world cannabis use behaviours among PWUD, including use with therapeutic or harm reduction intent, associated with beneficial outcomes (Mok et al. 2023, 2021; Lake et al. 2020; Fehr et al. 2025). For example, prior studies have found associations between cannabis use and reductions in opioid use and cravings (Reddon et al. 2023; Lake et al. 2019; Reiman et al. 2017), reductions in rates of injection drug use (Reddon et al. 2021, 2020, 2018), reduced odds of exposure to fentanyl (Socías et al. 2021; Hayashi et al. 2018), and improved retention in opioid agonist therapies (Socías et al. 2018; Raby et al. 2009). Our findings contribute to this growing body of evidence and demonstrate that intentional cannabis use to mitigate symptoms of unregulated opioid withdrawal may support reductions in opioid use, especially among people living with pain.

The findings in this study differ from those of Gorfinkel et al., who examined daily cannabis use and non-medical opioid use to assess potential substitution among people who use non-medical opioids (Gorfinkel et al. 2021). In that study, the odds of opioid use were approximately double on days when cannabis was also used, suggesting that cannabis did not function as a substitute for opioids in their study population (Gorfinkel et al. 2021). Although participant characteristics across studies were broadly similar, these divergent findings may reflect differences in study design and measurement. Specifically, Gorfinkel et al. assessed any cannabis use regardless of motivation and focused on same-day co-use, whereas the present study examined self-reported intentional cannabis use for opioid withdrawal management and self-reported changes in opioid use over broader periods. These approaches may capture distinct patterns of cannabis use, including general polysubstance use versus intentional therapeutic or harm reduction-oriented use.

As in previous studies, we detected an important role for chronic pain. There was a substantial prevalence of moderate to severe pain in our sample of PWUO (48.2%), consistent with prior estimates in similar populations (Voon et al. 2017, 2014; Lusted et al. 2013), which is likely not controlled through licit clinical means. Given the central role of chronic pain in unregulated opioid initiation, use, dependence, withdrawal, and relapse (Voon et al. 2018, 2015; Dahlman et al. 2017), the potential of cannabis to address pain-related symptoms may explain, at least in part, its role in facilitating reductions in unregulated opioid use. This is supported by prior research documenting associations between medical cannabis use and reductions in opioid medication doses among people receiving treatment for pain (Okusanya et al. 2020), as well as longitudinal research demonstrating a negative association between frequent cannabis use and unregulated opioid use among people who use drugs living with chronic pain (Lake et al. 2019).

Any possible benefits of cannabis use must be balanced by its potential risks, both acute (e.g., over-intoxication, accidents) and chronic (e.g., dependence), particularly among younger individuals or those with a personal or family history of psychosis, even if the potential nature and direction of effects of cannabinoids on psychosis remain unclear) (Walsh et al. 2017; Lake et al. 2020; Volkow et al. 2016; Ksir and Hart 2016). Similarly, people at high risk of fatal overdose through exposure to the unregulated drug supply might weigh their risk–benefit calculation differently. Importantly, cannabis is associated with markedly lower risks of morbidity and mortality compared to unregulated opioids, a conclusion supported by comparative drug harm assessments and epidemiological evidence (Lake et al. 2020; Nutt et al. 2010). Qualitative and ethnographic studies among PWUD further demonstrate that individuals’ perceptions of cannabis as a safer alternative align with these relative risk profiles (Paul et al. 2020; Ganesh et al. 2024; Beaugard et al. 2024; Valleriani et al. 2020). For example, in one study from the United States with interviews with people in recovery from drug dependence, all endorsed the idea that cannabis was a safer alternative to other drugs, pointing to its lack of contaminants, its legal status, and its inability to cause a fatal overdose (Beaugard et al. 2024).

Although consistent with the results of studies from our setting and others, these findings do not offer conclusive evidence supporting the use of cannabis for opioid withdrawal, which should be the subject of experimental study among humans. Nevertheless, even in the absence of evidence from controlled trials, policymakers could consider efforts to review and eliminate structural barriers to legal cannabis, including access and affordability, for PWUD using cannabis for therapeutic periods during the ongoing and unprecedented overdose crisis (Valleriani et al. 2020). All available evidence suggests that legal cannabis—from either the medical or non-medical cannabis regimes in Canada—remains largely inaccessible to PWUD, who continue to access cannabis through the unregulated market (Lake et al. 2020; Reddon et al. 2023). This is not only inequitable but also undermines the black letter public health goals of non-medical cannabis legalization in Canada’s *Cannabis Act* (Cannabis Act (S.C. 2018, c. 16) and the constitutional right of Canadians to access cannabis for medical purposes from a legal source (Parker 2000).

This study should be interpreted in the context of several limitations. First, participants in the VIDUS, ACCESS, and ARYS cohorts are not randomly selected, which may limit the generalizability of these findings to other settings. Second, given the cross-sectional study design, causality and the direction of associations cannot be established. Future research should incorporate more granular measures of opioid use, including quantity, to better capture the nuances of use patterns. Third, our reliance on self-reported substance use data introduces potential recall and social desirability bias. However, prior research has established that self-reported substance use patterns and behaviours among PWUD are valid and reliable when compared to biomarker assessments (Bharat et al. 2023; Darke 1998). In this study, participants were asked to reflect on perceived changes in opioid use during periods of cannabis use, which required participants to consider a connection between the frequency of use of two different substances. This may have introduced measurement error, including the potential for exaggerated or misattributed associations between cannabis use and opioid use. Alternative approaches, such as more frequent data collection (e.g., daily or weekly assessments), may provide more precise estimates of temporal relationships between cannabis use and opioid use. Finally, reductions in opioid use were assessed subjectively and in reference to periods of cannabis use. This measure reflects participants’ perceived reductions rather than objectively measuring changes and did not quantify the magnitude of reductions, nor or changes in actual opioid use frequency,as such, we are unable to determine whether reported reductions reflect clinically meaningful differences in opioid use. Moreover, the outcome measure did not include a parallel measure assessing perceived increases in opioid use during periods of cannabis use. As such, participants categorized as not reporting reductions may have experienced either no change or increases in opioid use, limiting our ability to characterize the full distribution of opioid use changes associated with cannabis use. Future studies should incorporate more granular and bidirectional measures of opioid use changes during periods of cannabis use.

## Conclusion

In summary, we observed that self-reported intentional use of cannabis to manage symptoms of unregulated opioid withdrawal was associated with self-assessed decreases in opioid use among individuals using unregulated opioids and cannabis, living with moderate to severe pain. Our results add to other evidence documenting cannabis use to mitigate the risks arising from the unregulated drug supply. While there is a need to estimate the risks and benefits of cannabis during opioid withdrawal through high-quality controlled trials, immediate efforts to improve access to legal cannabis—especially for structurally-marginalized PWUD at highest risk of overdose—may offer a step towards reducing the health and social harms of opioid use in Canada.

## Data Availability

The datasets used and/or analyzed during the current study are available from the corresponding author on a reasonable request.
